# Use of Social Media Data to Diagnose and Monitor Psychotic Disorders: Systematic Review

**DOI:** 10.2196/36986

**Published:** 2022-09-06

**Authors:** Alban Lejeune, Benoit-Marie Robaglia, Michel Walter, Sofian Berrouiguet, Christophe Lemey

**Affiliations:** 1 Unité de Recherche Clinique Intersectorielle Hôpital de Bohars Centre Hospitalier Régional Universitaire de Brest Bohars France; 2 Institut Polytechnique de Paris Palaiseau France; 3 Faculté de Médecine et Sciences de la Santé Université de Bretagne Occidentale Brest France; 4 Laboratoire de Traitement de l'Information Médicale Unité Mixte de Recherche 1101 Institut National de la Santé et de la Recherche Médicale Brest France; 5 Lab-STICC Unité Mixte de Recherche, Centre National de la Recherche Scientifique 6285, F-29238 École Nationale Supérieure Mines-Télécom Atlantique Bretagne Pays de la Loire Brest France

**Keywords:** schizophrenia, psychotic disorders, psychiatric disorders, artificial intelligence, AI, machine learning, neural network, social media

## Abstract

**Background:**

Schizophrenia is a disease associated with high burden, and improvement in care is necessary. Artificial intelligence (AI) has been used to diagnose several medical conditions as well as psychiatric disorders. However, this technology requires large amounts of data to be efficient. Social media data could be used to improve diagnostic capabilities.

**Objective:**

The objective of our study is to analyze the current capabilities of AI to use social media data as a diagnostic tool for psychotic disorders.

**Methods:**

A systematic review of the literature was conducted using several databases (PubMed, Embase, Cochrane, PsycInfo, and IEEE Xplore) using relevant keywords to search for articles published as of November 12, 2021. We used the PRISMA (Preferred Reporting Items for Systematic Reviews and Meta-Analyses) criteria to identify, select, and critically assess the quality of the relevant studies while minimizing bias. We critically analyzed the methodology of the studies to detect any bias and presented the results.

**Results:**

Among the 93 studies identified, 7 studies were included for analyses. The included studies presented encouraging results. Social media data could be used in several ways to care for patients with schizophrenia, including the monitoring of patients after the first episode of psychosis. We identified several limitations in the included studies, mainly lack of access to clinical diagnostic data, small sample size, and heterogeneity in study quality. We recommend using state-of-the-art natural language processing neural networks, called language models, to model social media activity. Combined with the synthetic minority oversampling technique, language models can tackle the imbalanced data set limitation, which is a necessary constraint to train unbiased classifiers. Furthermore, language models can be easily adapted to the classification task with a procedure called “fine-tuning.”

**Conclusions:**

The use of social media data for the diagnosis of psychotic disorders is promising. However, most of the included studies had significant biases; we therefore could not draw conclusions about accuracy in clinical situations. Future studies need to use more accurate methodologies to obtain unbiased results.

## Introduction

### Background

Schizophrenia is a chronic mental disease affecting 20 million people worldwide [1]. Although treatment with medicine and psychosocial support is effective, people with schizophrenia are less likely to seek treatment. According to the World Health Organization, efforts to transfer care from mental health institutions to the community need to be accelerated [2]. Currently, schizophrenia is a disease associated with high burden [3,4], and efforts should be taken to reduce this burden.

Artificial intelligence (AI) has emerged as a way to improve several medical tasks [5,6]. AI algorithms can identify patterns in a data set to generate diagnostic tools. In other medical disciplines, AI has already shown good accuracy for diagnostic purposes. It can match current diagnostic capabilities in some specific fields. In psychiatry, AI could be used for diagnostic purposes to support daily patient assessment or drug prescription [7]. AI has also been studied to improve diagnostic and classification capabilities [8]. Additionally, it has been used in suicide risk detection [9] and mood disorder diagnoses [10,11].

Despite encouraging results, it is still unclear how AI should be implemented in clinical practice. The potential of this technology is not yet fully understood. AI could be used to bring completely new tools into health care. We believe that social media might be used to create new diagnostic or monitoring tools. Indeed, AI requires a large database to extract a patient’s profile [12], and social media platforms provide very broad sources of information. People can disclose personal information on social media, including health-related information. Studies have used these data to identify broad human traits (such as intelligence or personality traits) [13]. Subtle features of everyday language could be analyzed to predict mental diseases [14-16]. Prior works showed that social media data can be used for risk classification associated with mental health diseases, such as suicide risk [17]. AI can be used to detect posts associated with mental illness [18]. Therefore, we chose to study the use of AI applied to social media because we believe it could become a brand-new tool in the care of patients with psychotic disorders.

### Focus on AI Technologies

AI can be used to perform several different tasks. Machine learning algorithms are generally classified as supervised or unsupervised learning. The main type of machine learning algorithms used in the included studies was supervised learning. Supervised learning algorithms allow patterns correlated to a result to be determined in a data set [12]. The supervised algorithms are separated into two categories: regression and classification. Classification algorithms allow data to be classified into separate categories. Patterns can be used to classify patients in a given group. Decision tree, support vector machine (SVM), and random forest can perform classification tasks. Regression algorithms are used to predict quantitative data. Logistic regression and LASSO (least absolute shrinkage and selection operator) regression are part of this class [6].

Artificial neural networks (NNs) are powerful AI tools built in reference to the cortical neural structure. They can perform supervised or unsupervised tasks. NNs are organized in a succession of layers, with each layer having its input on the output of the previous one. Information travels from the input neurons to the hidden layers before arriving at the outcome layer where the final decision is made [5]. In an NN, each layer functions differently. This type of AI requires significant computing power and large databases [19].

The most used machine learning algorithm in our study is the SVM [20]. The idea of this algorithm is to learn a linear separation (a hyperplane) of the data points to classify them. As there are infinite hyperplanes satisfying this condition, the SVM algorithm learns a hyperplane with the maximum margin, the maximum distance between the classes. However, as most real-life data sets cannot be linearly separated, the SVM uses what is called the “kernel trick.” This transformation projects the initial data points in a higher dimensional feature space where the new is linearly separable. However, there are many limitations to consider when using the SVM: (1) finding a good kernel function is difficult in practice, (2) training is time-consuming on large data sets, and (3) the model is very sensitive to the hyperparameters.

Overfitting means fitting an AI model on data noise or error instead of the actual relationship. It represents one of the limitations of AI. Overfitting is either due to having a small data sample or too many parameters compared to the data [12]. Cross-validation is one of the techniques used to reduce overfitting. With this technique, the data set is split into several groups that are themselves divided into training data and validation data. Therefore, for each group, the statistical model is trained and then validated by a different data set. This technique reduces the risk of having an overoptimistic estimate [21]. Other techniques such as the dropout rate are also used to reduce overfitting. Dropout is a regularization technique for NNs to reduce overfitting and improve generalization [22]. The idea is to randomly ignore neurons (and their connections) from the NN during training. Thus, as the NN architecture is changing at every inference, the same input data can produce a different output. The intuition is that it forces the units to be less codependent and more robust. The main difference between cross-validation and dropout lies in the source of randomness; in cross-validation, the data are randomly split into training and validation sets whereas in dropout, the neural units are randomly discarded.

We have explained the machine learning parameters used in this paper in Multimedia Appendix 1 [23-25].

### Prior Work and Goal of This Study

Currently, the diagnosis of psychotic disorders can be subjected to delay. These delays can vary depending on where the patient lives. When the patient remains untreated with psychotic symptoms, there can be important social consequences, including a risk of violence in some cases [26]. The duration of untreated psychosis could have a significant impact on the patient’s psychosocial condition. Early detection and treatment could help improve the care of patients with psychotic disorders [27]. Later, during the evolution of the disease, being able to diagnose a relapse sooner could have significant impact on the patient’s quality of life and reduce caregiver burden.

AI has been studied for the diagnosis of several psychiatric conditions, including schizophrenia. A Korean team used machine learning to identify patterns on CT (computed tomography) scans and classify patients [28]. One of the most robust studies on the classification of psychotic disorders using machine learning comes from an American team [8] that used a clustering algorithm to build 3 biotypes using clinical data and laboratory measures. Neuroimaging and social functioning measures were used for validation. Beyond the main initial goal, this study showed that several new sources of data can be used to improve diagnostic capabilities. In our study, we examined social media data as a new source of data. The symptoms of schizophrenia are very broad, and some of these symptoms could impact patients’ social media activities. For example, we can hypothesize that delusion and disorganized speech or behavior could be seen on social media posts. Alterations in language are being increasingly studied in schizophrenia [15,29] and could be used for the detection of psychotic disorders on social media.

Several studies have shown the capabilities of AI in identifying mental health diseases on social media, with most of them published in the last few years. One brief report [30] reviewed the literature available until December 2020. We hypothesize that social media data could be used to follow patients with schizophrenia or patients at risk of psychosis to identify the first psychotic episode or a relapse of psychotic disorders sooner. Patients could have access to care before psychotic symptoms overtake their social functioning capabilities. Thus, we could reduce the burden of schizophrenia.

### Objective of This Review

The objective of our study is to analyze prior works on the use of social media data with machine learning to diagnose a psychotic episode. The diagnostic capabilities would be studied in a broad sense, including the diagnosis of relapse. Therefore, we performed a systematic review of the literature and critically evaluated the included articles and their methodology.

## Methods

### Search Strategy

We used the PRISMA (Preferred Reporting Items for Systematic Reviews and Meta-Analyses) criteria to identify, select, and critically assess relevant studies while minimizing bias. We searched the bibliographic databases PubMed, Embase, Cochrane, PsycInfo, and IEEE Xplore for studies published until November 12, 2021. We based the keywords list on 3 fields: schizophrenia, AI, and social networks. The search strategy is described in Multimedia Appendix 2. To limit the selection bias, we did not apply any restriction in terms of population. Papers found by any other means were included if they met the inclusion criteria. Studies that were not published in English were excluded.

### Study Selection

We included clinical trials and observational studies. The primary objective was to include studies using AI to identify users with a psychotic disorder on social media. Given the low number of published studies on this subject, studies related to any psychotic disorder were included. Studies were selected by 2 independent authors. We excluded studies using social media posts as control data to study language alterations in schizophrenia.

### Data Collection Process

Data were extracted from each paper independently using a standard form by 1 reviewer. The following information was collected: the main author’s name and country of origin, year of publication, population, social media and technology used, features, inclusion and exclusion criteria, main objective, method, main endpoint, results, and main limitations.

### Synthesis Method

The results of the selected studies will be presented as graphs and tables. Machine learning studies often use different parameters. The graphs will group studies using similar parameters. No secondary analysis of statistical data will be performed. The results presented will comprise only those presented by the authors of the included studies. The results will be presented with the parameters used in the articles. The machine learning parameters used in this paper are explained in Multimedia Appendix 1.

### Risk of Bias and Quality Assessment

The quality of the included studies will be assessed using the PRISMA certainty tool. This research will be conducted using the PRISMA checklist (Multimedia Appendix 3). The machine learning methodology of the articles will be evaluated by an author experienced in AI. Risk of bias will be critically assessed by all the authors.

## Results

### Flowchart

**Figure 1:**
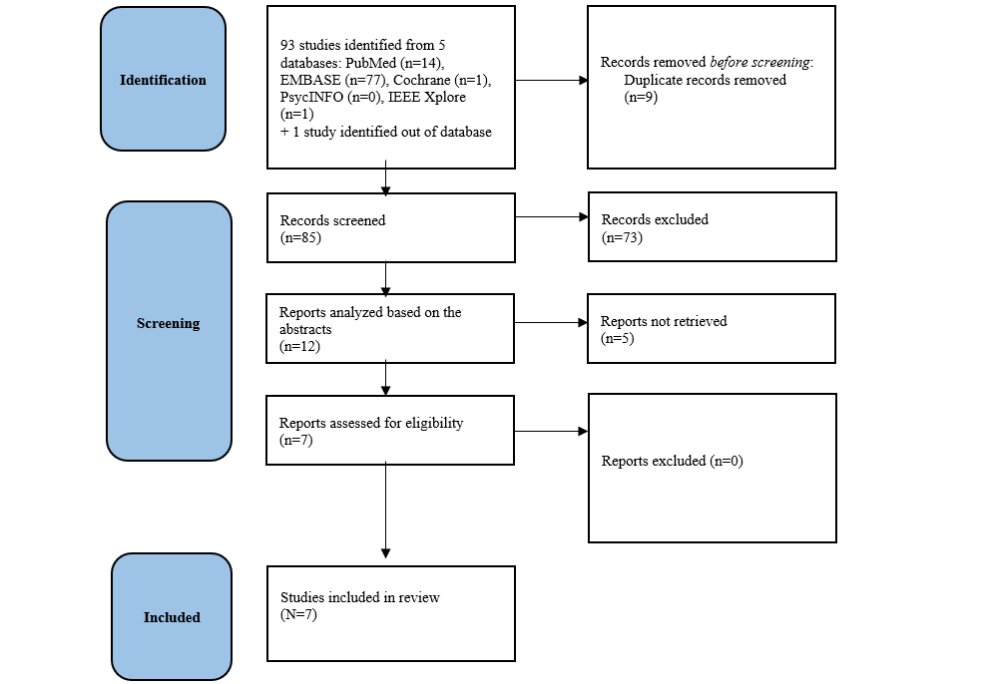
PRISMA (Preferred Reporting Items for Systematic Reviews and Meta-Analyses) flowchart outlining the study selection process.

We developed a PRISMA flowchart summarizing the steps of the review (Figure 1). The initial search yielded 93 studies. Based on the titles and abstracts, we excluded 78 studies. We downloaded the remaining studies for full-text review and included the 7 studies that matched the inclusion criteria.

### Authors, Year of Publication, and Country of Origin

The included studies were conducted in the United States (5/7) and Korea (2/7) and were published between 2015 and 2021. Birnbaum and colleagues conducted 3 of the 7 included studies.

### Study Design, PRISMA Quality Assessment, and Sample Size

The included studies had a retrospective design. The quality of the studies was assessed using the PRISMA criteria (Table 1 and Multimedia Appendix 4). The quality of the 7 studies was heterogeneous, with a mean PRISMA score of 32.3. Risk of bias varied across the studies. The main bias was a classification bias in 5 of the 7 studies not using a clinical diagnosis. The sample sizes were mostly small, with 6 of the 7 studies having a sample size smaller than 5392 participants. The samples size varied between 51 and 265,396 participants.

**Table 1:** PRISMA (Preferred Reporting Items for Systematic Reviews and Meta-Analyses) quality scores of the included studies.

Study	PRISMA quality score^a^
Birnbaum et al [31], 2017, United States	31
Kim et al [32], 2020, Korea	31
Birnbaum et al [33], 2019, United States	37
Birnbaum et al [34], 2020, United States	36
McManus et al [35], 2015, United States	29
Bae et al [36], 2021, Korea	36
Mitchell et al [37], 2015, United States	29

^a^The higher the score, the better the overall quality.

### Social Media and Technologies Used

Several AI technologies have been used (Table 2). The 2 most commonly used algorithms were SVM and random forest. None of the included studies used data augmentation. Cross-validation techniques to prevent overfitting were used in 5 studies. One study used a dropout rate of 0.25 to prevent overfitting. The social media platforms the studies used were Facebook, Twitter, and Reddit. The studies used mainly linguistic features, as well as some activity-related features (Table 3).

**Table 2:** Information extracted from the included studies.

Authors, year, and country	Overview and inclusion criteria	Objective	Method	Social media and AI^a^ technology	Outcome	Main limitations
Birnbaum et al [31], 2017, United States	Users with a self-disclosed diagnosis of schizophrenia on Twitter between 2012 and 2016. Authors randomly selected 671 users diagnosed with schizophrenia from the primary data set. The control group comprised a random sample of Twitter users collected from individuals without any mentions of “schizophrenia” or “psychosis” in their timeline.	To explore the utility of social media as a viable diagnostic tool in identifying individuals with schizophrenia	Twitter profiles from the training data set were reviewed by 2 physicians to determine the probability of belonging to a patient with schizophrenia. The users were then classified into 3 groups: “yes,” “maybe,” or “no.” The machine learning model was then tested on unseen data of 100 users and its results were compared to those of the 2 physicians.	Twitter. Several algorithms including the Gaussian naïve Bayes (NB), random forest (RF), logistic regression (LR), and support vector machine (SVM) were trained. The best performing algorithm on cross-validation was selected (RF) using 10-fold-cross-validation.	RF yielded an area under the curve (AUC) of 0.88.	The research team only had access to publicly available Twitter profiles. The clinical diagnosis of the included users was unknown.
Kim et al [32], 2020, Korea	Data from 228,060 users with 488,472 posts from January 2017 to December 2018 were employed for the analysis.	Aimed to answer the following question: Can we identify whether a user's social media post can be associated with a mental illness?	Collection of post data on mental health–related subreddit groups. The study collected information from 248,537 users, who wrote 633,385 posts in the 7 subreddits. After removal of deleted users and posts, 488,472 posts were analyzed. Authors created 6 models for each mental disorder. Each model was created with the posts of the associated subreddit group.	Reddit. Extreme gradient boosting (XGBoost) and convolutional neural network (CNN) were employed. A dropout rate of 0.25 was used to prevent overfitting issues.	In the schizophrenia subreddit (r/schizophrenia), accuracies of XGBoost and CNN were 86.75% and 94.33%, respectively.	The clinical diagnosis of included subjects was unknown.
Birnbaum et al [33], 2019, United States	Participants aged 15 to 35 years diagnosed with a primary psychotic disorder were screened for eligibility. Recruitment occurred between March 2016 and December 2018, and 51 of the included participants had a recent onset of psychosis.	To identify and predict early relapse warning signs in social media activity collected from a cohort of individuals receiving psychiatric care for schizophrenia and other primary psychotic disorders	The authors collected 52,815 Facebook posts across 51 participants with a recent onset of psychosis and applied anomaly detection to explore linguistic and behavioral changes associated with psychotic relapse.	Facebook. Three 1-class SVM models for 3 different data configurations (3 different time periods: 1 month, 2 months, and 3 months). The 1-month period showed the highest specificity, which led to an ensemble 1-class SVM algorithm.	The ensemble model had the highest specificity (0.71) but low sensitivity (0.38). The 3-month model had good sensitivity (0.9) but low specificity (0.04).	Monthly periods of relative health and relative illness were characterized. The illness trajectory of psychotic disorder does not fall only into 2 distinct categories, as the symptoms can fluctuate over time.
Birnbaum et al [34], 2020, United States	A total of 3,404,959 Facebook messages and 142,390 images across 223 participants with schizophrenia spectrum disorders (SSD), mood disorders (MD), and healthy volunteers (HV) were collected. Participants aged between 15 and 35 years were recruited from Northwell Health’s psychiatry department.	To evaluate whether it was possible to distinguish among SSD, MD, and HV based on Facebook data alone.	The authors analyzed features uploaded up to 18 months before the first hospitalization using machine learning and built classifiers that distinguished SSD and MD from HV as well as SSD from MD.	Facebook. RF and 5-fold cross-validation were used.	Classification achieved AUC values of 0.77 (HV vs MD), 0.76 (HV vs SSD), and 0.72 (SSD vs MD).	Data from Facebook were retrospectively collected.
McManus et al [35], 2015, United States	The cohort contained Twitter users who self-identified as having schizophrenia (cases) and users who did not self-identify as having any mental disorder (controls), with 96 cases and 200 controls. A user was defined as a case if 2 or more of the following held true: The user self-identifies in the user description; the user self-identifies in their status updates; the user follows @schizotribe, a known Twitter community of users with schizophrenia.	To distinguish individuals with schizophrenia from control individuals using Twitter data	To distinguish Twitter users with schizophrenia from controls, the authors extracted a set of features from each user's profile and posting history (28 numerical features).	Twitter. Several models: NB, artificial neural networks (ANNs), and SVMs. 5-fold cross validation on the training data. In addition to the raw feature vectors, the authors tested 2 transformations of the feature vectors for each of the models: log scaling of the delay between tweets and principal component analysis (PCA).	The best performing model was an SVM with PCA-transformed features (accuracy of 0.893). The 2 best performing models based on the F1 score involved PCA-transformed features.	Users self-identified as patients with schizophrenia.
Bae et al [36], Korea, 2021	A large corpus of social media posts was collected from web-based Reddit subcommunities for schizophrenia (n= 13,156) and control groups (n=247,569) comprising non-mental health–related subreddits (fitness, jokes, meditation, parenting, relationships, and teaching).	To determine whether machine learning could be effectively used to detect signs of schizophrenia in social media users by analyzing their social media texts	Authors collected posts from subreddit. They only included original posts and excluded the comments. They collected titles and bodies of posts along with user IDs. This resulted in 60,009 original schizophrenia posts from 16,462 users as well as 425,341 posts of the control group from 248,934 users.	Reddit. Posts from the control group were randomly downsampled to create a balanced data set (n= 13,156 posts for each group). The authors evaluated 4 different algorithms, namely SVM, LR, NB, and RF, with 10-fold cross-validation.	AUC values were as follows: RF 0.97, SVM 0.91, LR 0.9, and NB 0.87	The authors do not have evidence that users of r/schizophrenia are clinically diagnosed.
Mitchell et al [37], United States, 2015	A corpus of users diagnosed with schizophrenia was collected from publicly available Twitter data, including 174 users with an apparently genuine self-stated diagnosis of a schizophrenia-related condition. Random Twitter users were included as the control, and there were equal numbers of users with schizophrenia and community controls.	To examine how linguistic signals may aid in identifying and getting help to people with schizophrenia	Each self-stated diagnosis included in this study was examined by an author to verify that it appeared to be a real statement of a schizophrenia diagnosis, excluding jokes, quotes, or disingenuous statements. For each user, the authors obtained a set of their public Twitter posts via the Twitter application programming interface, collecting up to 3200 tweets.	Twitter. The authors used 10-fold cross-validation and 2 machine learning methods, namely SVM and maximum entropy.	The SVM model reached an 82.3% accuracy.	Clinical diagnosis was unknown.

^a^AI: artificial intelligence.

**Table 3:** Features used in the included studies.

Authors, year, and country	Features
Birnbaum et al [31], 2017, United States	The authors employed feature scaling to standardize the range of features. The LIWC^a^ features were within a normalized range of 0 to 1. The n-gram features represented frequency counts that required standardization. The min-max rescaling technique was used to scale the n-gram features to the range of 0 to 1. They employed feature selection methods to eliminate noisy features. The filter method was used where features are selected on the basis of their scores in various statistical tests for their correlation with the outcome variable. Adopting the ANOVA F test reduced the feature space from 550 features to k – best features (where k=350) by removing noisy and redundant features.
Kim et al [32], 2020, Korea	The natural language toolkit was implemented in Python (Python Software Foundation) to tokenize users’ posts and filter frequently employed words (stop words). Porter stemmer (a tool used to explore word meaning and source) was employed on the tokenized words to convert a word to its root meaning and to decrease the number of word corpora.
Birnbaum et al [33], 2019, United States	Facebook timeline data grounded in the symptomatic and functional impairments associated with psychotic disorders were used. These include 3 types of features. The first was word usage and psycholinguistic attributes related to affective, social, and personal experiences. The second included linguistic structural attributes, such as complexity, readability, and repeatability related to thought organization and cognitive abilities. The third comprised web-based activities relating to social functioning and diurnal patterns (friending, posting, and check-ins).
Birnbaum et al [34], 2020, United States	Image and linguistic features were used.
McManus et al [35], 2015, United States	Features for describing emoticon use and schizophrenia-related words were used. The authors used the natural language toolkit in Python to perform tokenization and lemmatization, before extracting textual features and NumPy for generating the final numeric feature vectors. The final 28 numerical features included the number of Twitter followers, number of followed users, proportion of tweets using schizophrenia-related words, emoticon usage, posting time of day, and posting rate. Two transformations of the feature vectors for each of the models were used: log scaling of the delay between tweets and principal component analysis.
Bae et al [36], Korea, 2021	The linguistic features were extracted using the LIWC package and the *liwcalike* function from the *quanteda* package. Structural and psychological components of the text based on psychometrically validated dictionary, word stems, and emotions assigned to a range of categories were assessed. There were 22 LIWC features for each post: linguistic processes (word count and words more than 6 letters), function words (personal pronouns, first-person singular, first-person plural, second person, third-person singular, third-person plural, and impersonal pronouns), time orientations (past focus, present focus, and future focus), and psychological processes (positive emotion, negative emotion, anger, fear, joy, disgust, sadness, anticipation, trust, and surprise). Linguistic features between the schizophrenia and the control (nonschizophrenia) groups were compared. The D’Agostino and Pearson’s test (α=.05) were conducted to test whether each of the linguistic features was normally distributed. As data followed a normal distribution, a 2-tailed *t* test was performed to determine whether the linguistic features differed between groups. The threshold of statistical significance was adjusted using the false discovery rate method to correct for multiple comparisons, with *P*<.05 in all cases.
Mitchell et al [37], United States, 2015	All natural language processing features were either automatically constructed or unsupervised, meaning that no manual annotation is required to create them. It is important to note that although these features were inspired by the literature on schizophrenia, they were not direct correlates of standard schizophrenia markers. The authors used the following methods to extract features: perplexity (ppl), Brown-Cluster Dist, LIWC, CLM^b^, LIWC+CLM, LDA^c^ Topic Dist (TDist), CLM+TDist+BDist+ppl, CLM+TDist, and LIWC+TDist. The authors used the LIWC approach to map the words to psychological concepts as well as open-vocabulary approaches such as LDA, Brown clustering, CLM, or perplexity in order to extract features from the corpus in an unsupervised manner. In particular, the LDA algorithm learns a probability distribution over topics for each document. The Brown clustering is a hierarchical clustering algorithm that groups words that occur in similar contexts. Regarding the CLM method, the idea is to assign a probability to a sequence of words (n-grams). In the paper, the authors used a sequence of 5 characters (5-grams). Finally, perplexity is a measurement of how predictable the language is. We expect a high perplexity score for a user using a noncoherent language.

^a^LIWC: linguistic inquiry and word count.

^b^CLM: character language model.

^c^LDA: latent Dirichlet allocation.

### Study Objectives and Algorithm Performance

#### Main Results

Most studies aimed to identify users with schizophrenia on social media. One study aimed to identify and predict early relapse after hospitalization for schizophrenia [33].

The results were informed by multiple parameters, including the area under the curve (AUC), accuracy, as well as sensitivity and specificity. The AUC of the included studies ranged from 0.76 to 0.97 (Table 4), which is considered to be good to excellent. However, only 1 of these studies [34] used data from clinically diagnosed patients, obtaining an AUC of 0.76. The studies whose results were informed by the accuracy parameter obtained an accuracy ranging from 81% to 96%. One study [33] reported results obtained using predictive models with a sensitivity/specificity couple (Table 5). This study sought to identify and predict relapse of schizophrenia. The authors collected Facebook data from a small sample of patients diagnosed with schizophrenia who had a relapse in the following months. They used these data to build a machine learning model that could be used to analyze the patients’ data in real time. They obtained several sensitivity/specificity couples. The 3-month ensemble model showed good sensitivity (90%) although the specificity was low (40%). This is an example of the unique tools that could be developed using AI. A high-sensitivity tool could allow physicians to detect a relapse earlier and offer timely care to their patients. The 1-month model had a high specificity (0.71) but low sensitivity (0.38).

**Table 4:** Performance of the different algorithms in terms of the area under the curve.

Study	Support vector machine	Random forest	Logistic regression	Naïve Bayes
Birnbaum et al [31], 2017, United States	—^a^	0.88	—	—
Birnbaum et al [34], 2020, United States	—	0.76	—	—
Bae et al [36], Korea, 2021	0.91	0.97	0.90	0.87

^a^Not applicable.

**Table 5:** Performance of the different algorithms in terms of accuracy and sensitivity/specificity.

Study	Accuracy (%)	Sensitivity/specificity (%)
Birnbaum et al [31], 2017, United States	81 (RF^a^)	—^b^
Kim et al [32], 2020, Korea	86.75 (XGB^c^), 94.33 (CNN^d^)	—
Birnbaum et al [33], 2019, United States	—	38/71, 90/40 (SVM^e^)
McManus et al [35], 2015, United States	89.3 (SVM)	—
Bae et al [36], Korea, 2021	86 (NB^f^), 89 (LR^g^), 91 (SVM), 96 (RF)	—
Mitchell et al [37], 2015, United States	82.3 (SVM)	—

^a^RF: random forest.

^b^Not applicable.

^c^XGB: extreme gradient boosting.

^d^CNN: convolutional neural network.

^e^SVM: support vector machine.

^f^NB: naïve Bayes.

^g^LR: logistic regression.

#### Data Used

Most of the studies did not have access to clinical diagnostic or health data. Instead, they used evaluations of users’ profiles by psychiatrists to access which user could be classified as having schizophrenia. The included studies used the content of the posts to train and test the models. They also used activity-derived markers such as friending, check-ins, and the number of followers. Used features were chosen to represent the symptoms of schizophrenia described in the literature and were focused on identifying disorganized symptoms and cognitive abilities (Table 2).

## Discussion

### Principal Results

On a statistical basis, the included studies reported good to excellent performance. Indeed, many of the metrics they reported on are at the top of their respective ranges (AUC, accuracy, sensitivity, and specificity). However, accuracy has high chances of being biased, as most studies did not have access to clinical diagnostic data to train the models. Most studies used the evaluations of social media profiles by trained physicians to classify patients into different groups. Thus, we cannot reach any conclusions regarding the performance of AI in detecting patients with schizophrenia on social media. Moreover, 2 of the included studies that did have access to clinical diagnostic data [33,34] showed the most conservative results (Tables 3 and 4). The included studies were heterogeneous, and some of them introduced interesting new perspectives. After the first psychotic episode, AI and social media could be used to monitor the clinical state of the patients and detect a relapse sooner. This strategy has been studied by Birnbaum et al [33]. We also hypothesize that it could be useful with cohorts of ultra-high-risk patients. Social media provides a constant flow of data, which could in theory allow for the monitoring of large patient cohorts and detect early signs of a psychotic episode. This tool could be integrated in the care of these patients with their consent.

### Critical Assessment of the Machine Learning Methodology in the Included Studies

The problem we are considering in this review is a binary classification problem (whether a user profile on social media indicates schizophrenia) mainly based on textual data (the user’s posts). In this section, we introduce the key challenges that we need to tackle given the collected data, analyze the methodology used in the literature, and present our machine learning methodology to solve this problem.

### Key Challenges in Performing Statistical Studies

The first major obstacle statisticians need to tackle is the imbalanced data set [38]. Indeed, in the included studies, the number of included controls is several times higher than the included cases. Thus, a “naïve” binary classifier (random forest, SVM, logistic regression, etc) should not be used. It would tend to overestimate the dominant class over the minor one. Moreover, the small sample size in the included studies suggests that there is a chance that the probability distribution in clinical practice is different than the one in the training set used for the experiments.

The second challenge imposed by the data structure is the textual data. Indeed, unlike most problems, we must deal with unstructured data, as opposed to structured data where the features are well organized in a table. Here, the data we consider include text (posts of the users), and it is unstructured. Therefore, natural language processing (NLP) techniques are called for to extract relevant features to run a machine learning classifier. This is often a delicate stage, as researchers often introduce an inductive bias when they decide which features to extract from the data. State-of-the art NN algorithms like the BERT (Bidirectional Encoder Representations from Transformers) algorithm [39] can automatically extract features from textual data in a “pretrained stage.” The algorithm is then trained to perform the desired classification task during the “fine-tuned stage.”

### Analysis of the Methodology Used in Previous Works

Most previous works introduced in the last section extract their features using NLP modules such as the natural language toolkit [32,35], the linguistic inquiry and word count package [36], or even older methods like the n-gram [31]. The major drawback of these “bag-of-words” techniques [40] or term frequency–inverse document frequency methodologies [41] is that they often vectorize the textual data only based on the words (and their statistics in the sentences) without accounting for grammar and semantic relations between them and their context. Thus, these feature extraction methods fall short when capturing semantic or syntactic information or the sentiments of words [42].

### Perspectives for Future Studies: Our Recommendations for Machine Learning Methodology

Based on the aforementioned challenges and the limitations of previous works, we present in this section our approach to identify patients with schizophrenia based on social media activity.

First, we need to address the imbalanced data set problem. Among the numerous approaches, we selected one of the most used methods in practice called SMOTE (Synthetic Minority Oversampling Technique) [43]. The idea is to balance the data set by creating synthetic samples from the minority class so that both classes become more balanced. Specifically, SMOTE selects an example from the minority class and its k neighbors (typically k=5) and creates a synthetic example as the convex combination of these 2 data points. This procedure can produce as many synthetic samples for the minority class as needed, and it guarantees that these created examples are realistic, as they are close to the existing ones in the feature space.

However, for this data augmentation technique to work, the feature space needs to be continuous, which is not the case in textual data. To alleviate this issue, we need to use word embeddings. In NLP, word embedding is a continuous representation of a word that encodes the meaning of the word in a feature space, and it is usually a real-valued vector [44]. Thus, 2 words with close meanings like “ill” and “disease” will be closer in the feature space than 2 words like “ill” and “car.” Nevertheless, the remaining challenge is to create a relevant embedding space. A popular method in NLP is to use a pretrained language model like BERT [39]. This algorithm has been pretrained by Google on the concatenation of the two largest data sets: BookCorpus [45], gathering 11,038 unpublished books, and English Wikipedia, gathering 6,427,217 articles. These high-quality embeddings not only allow us to use the SMOTE method to augment the minority class, but they also allow us to represent the textual data in an informative feature space. The latter will be used as the input to our classification algorithm.

Finally, we need to define the binary classifier for our problem. As we have already used the BERT algorithm to create the word embeddings of the posts, the natural approach would be to use it as a binary classifier as well. To do so, we need to “fine-tune” it using our own data set by adding a linear layer to the existing NN architecture. This approach to text classification has demonstrated state-of-the-art results on 8 widely studied text classification data sets [46].

### Limitations of This Study

We performed a systematic review of studies using machine learning to identify schizophrenia on social media. Based on our hypothesis, the main limitation of our review is the small number of included studies. When submitting this paper for publication, the published studies on this subject were limited and we were not aware of any new study that met the inclusion criteria. Our review aimed to evaluate the potential of this technology as a new tool for the care of patients with schizophrenia. Therefore, we used broad inclusion criteria to include more papers. The included studies do not have the same objective and thus, their results cannot be compared. However, they describe the various uses of this technology.

Most of the studies (5/7) did not use clinical diagnostic data. Instead, they used evaluations of the mental states of the included subjects based on their public profile history and the contents of their posts. It is unlikely that this method is efficient in accurately identifying patients with schizophrenia. Future studies should use health data including medical diagnostic data to develop an accurate model.

Furthermore, some of the included studies showed limitations in their methodology and choice of machine learning algorithms. We analyzed these limitations and used them to propose recommendations for future projects.

### Ethical Reflection and Privacy

Machine learning tools could be useful in several ways to improve the care of patients. We could monitor the social media activity of patients to detect psychotic relapses sooner. These tools could also be used to detect the first psychotic episode sooner in patients monitored for high risk of psychosis.

The use of machine learning to predict mental health disease raises ethical questions. In what context should we use such tools? Patients would need to comply with the use of these tools and their data in their care. The use of machine learning would be appropriate only if patients consent to it. Furthermore, many countries are establishing a regulatory framework on AI usage [47,48]. AI tools will have to comply with regulation laws to be used in a clinical setting.

In particular, machine learning algorithms need to be trained on massive amounts of unbiased data. To prevent third parties from using these technologies for other purposes, ensuring the safety of medical data is essential.

### Conclusions

AI brings new perspectives in research on schizophrenia. It could be used to monitor the clinical condition of patients at risk of psychosis or to detect relapses of schizophrenia by observing patients on social media. There are currently only a few studies published on this subject, and most of them do not accurately estimate the potential of this technology. However, this technology could become a new tool in the care for patients with schizophrenia, ultimately reducing the burden on caregivers. It should be developed and used in accordance with ethical and legal frameworks.

## Appendix

Multimedia Appendix 1 Machine learning parameters.

Multimedia Appendix 2 Search strategy.

Multimedia Appendix 3 PRISMA (Preferred Reporting Items for Systematic Reviews and Meta-Analyses) checklist.

Multimedia Appendix 4 A summary of the included studies and the PRISMA (Preferred Reporting Items for Systematic Reviews and Meta-Analyses) certainty assessment.

## Abbreviations

AI: artificial intelligence
AUC: area under the curve
CT: computed tomography
LASSO: least absolute shrinkage and selection operator
NLP: natural language processing
NN: neural network
PRISMA: Preferred Reporting Items for Systematic Reviews and Meta-Analyses
SMOTE: Synthetic Minority Oversampling Technique
SVM: support vector machine

## References

[1] GBD 2017 Disease and Injury Incidence and Prevalence Collaborators (2018). Global, regional, and national incidence, prevalence, and years lived with disability for 354 diseases and injuries for 195 countries and territories, 1990-2017: a systematic analysis for the Global Burden of Disease Study 2017. Lancet.

[2] (2022). Schizophrenia. World Health Organization.

[3] Charlson F, Ferrari A, Santomauro D, Diminic Sandra, Stockings Emily, Scott James G, McGrath John J, Whiteford Harvey A (2018). Global Epidemiology and Burden of Schizophrenia: Findings From the Global Burden of Disease Study 2016. Schizophr Bull.

[4] Cloutier M, Sanon Aigbogun M, Guerin A, Nitulescu R, Ramanakumar AV, Kamat SA, DeLucia M, Duffy R, Legacy SN, Henderson C, Francois C, Wu E (2016). The economic burden of schizophrenia in the United States in 2013. J Clin Psychiatry.

[5] Sidey-Gibbons JAM, Sidey-Gibbons CJ (2019). Machine learning in medicine: a practical introduction. BMC Med Res Methodol.

[6] Deo RC (2015). Machine learning in medicine. Circulation.

[7] Lin E, Lin C, Lane H (2020). Precision psychiatry applications with pharmacogenomics: artificial intelligence and machine learning approaches. Int J Mol Sci.

[8] Mothi SS, Sudarshan M, Tandon N, Tamminga C, Pearlson G, Sweeney J, Clementz B, Keshavan MS (2019). Machine learning improved classification of psychoses using clinical and biological stratification: Update from the bipolar-schizophrenia network for intermediate phenotypes (B-SNIP). Schizophr Res.

[9] Lejeune A, Le Glaz A, Perron P, Sebti J, Baca-Garcia E, Walter M, Lemey C, Berrouiguet S (2022). Artificial intelligence and suicide prevention: a systematic review. Eur Psychiatry.

[10] Ma Y, Ji J, Huang Y, Gao H, Li Z, Dong W, Zhou S, Zhu Y, Dang W, Zhou T, Yu H, Yu B, Long Y, Liu L, Sachs G, Yu X (2019). Implementing machine learning in bipolar diagnosis in China. Transl Psychiatry.

[11] Gao S, Calhoun VD, Sui J (2018). Machine learning in major depression: from classification to treatment outcome prediction. CNS Neurosci Ther.

[12] Mooney SJ, Pejaver V (2018). Big data in public health: terminology, machine learning, and privacy. Annu Rev Public Health.

[13] Mori K, Haruno M (2021). Differential ability of network and natural language information on social media to predict interpersonal and mental health traits. J Pers.

[14] Rezaii N, Walker E, Wolff P (2019). A machine learning approach to predicting psychosis using semantic density and latent content analysis. NPJ Schizophr.

[15] Le Glaz A, Haralambous Y, Kim-Dufor D, Lenca P, Billot R, Ryan TC, Marsh J, DeVylder J, Walter M, Berrouiguet S, Lemey C (2021). Machine learning and natural language processing in mental health: systematic review. J Med Internet Res.

[16] Tariq S, Akhtar N, Afzal H, Khalid S, Mufti MR, Hussain S, Habib A, Ahmad G (2019). A novel co-training-based approach for the classification of mental illnesses using social media posts. IEEE Access.

[17] Ophir Y, Tikochinski R, Asterhan C, Sisso I, Reichart R (2020). Deep neural networks detect suicide risk from textual facebook posts. Sci Rep.

[18] Gkotsis G, Oellrich A, Velupillai S, Liakata M, Hubbard TJP, Dobson RJB, Dutta R (2017). Characterisation of mental health conditions in social media using informed deep learning. Sci Rep.

[19] Kriegeskorte N, Golan T (2019). Neural network models and deep learning. Curr Biol.

[20] Hearst M, Dumais S, Osuna E, Platt J, Scholkopf B (1998). Support vector machines. IEEE Intell Syst Appl.

[21] Bey R, Goussault R, Grolleau F, Benchoufi M, Porcher R (2020). Fold-stratified cross-validation for unbiased and privacy-preserving federated learning. J Am Med Inform Assoc.

[22] Srivastava N, Hinton G, Krizhevsky A, Sutskever I, Salakhutdinov R (2014). Dropout: a simple way to prevent neural networks from overfitting. JMLR.

[23] Mandrekar Jayawant N (2010). Receiver operating characteristic curve in diagnostic test assessment. J Thorac Oncol.

[24] Sasaki Y (2007). The truth of the F-measure.

[25] Kaggle.

[26] Látalová K (2014). Violence and duration of untreated psychosis in first-episode patients. Int J Clin Pract.

[27] Albert N, Weibell MA (2019). The outcome of early intervention in first episode psychosis. Int Rev Psychiatry.

[28] Jo YT, Joo SW, Shon S, Kim H, Kim Y, Lee J (2020). Diagnosing schizophrenia with network analysis and a machine learning method. Int J Methods Psychiatr Res.

[29] de Boer JN, Brederoo SG, Voppel AE, Sommer IE (2020). Anomalies in language as a biomarker for schizophrenia. Curr Opin Psychiatry.

[30] Feldman J, Hamlyn A, Rice T (2021). Social media in screening and monitoring for early intervention in psychosis. Schizophr Res.

[31] Birnbaum ML, Ernala SK, Rizvi AF, De CM, Kane JM (2017). A collaborative approach to identifying social media markers of schizophrenia by employing machine learning and clinical appraisals. J Med Internet Res.

[32] Kim J, Lee J, Park E, Han J (2020). A deep learning model for detecting mental illness from user content on social media. Sci Rep.

[33] Birnbaum ML, Ernala SK, Rizvi AF, Arenare E, R Van Meter A, De Choudhury M, Kane JM (2019). Detecting relapse in youth with psychotic disorders utilizing patient-generated and patient-contributed digital data from Facebook. NPJ Schizophr.

[34] Birnbaum ML, Norel R, Van Meter A, Ali AF, Arenare E, Eyigoz E, Agurto C, Germano N, Kane JM, Cecchi GA (2020). Identifying signals associated with psychiatric illness utilizing language and images posted to Facebook. NPJ Schizophr.

[35] McManus K, Mallory EK, Goldfeder RL, Haynes WA, Tatum JD (2015). Mining Twitter data to improve detection of schizophrenia. AMIA Jt Summits Transl Sci Proc.

[36] Bae YJ, Shim M, Lee WH (2021). Schizophrenia detection using machine learning approach from social media content. Sensors (Basel).

[37] Mitchell M, Hollingshead K, Coppersmith G (2015). Quantifying the Language of Schizophrenia in Social Media. Proceedings of the 2nd Workshop on Computational Linguistics and Clinical Psychology.

[38] Garcia E, Haibo He (2009). Learning from imbalanced data. IEEE Trans Knowl Data Eng.

[39] Devlin J, Chang M, Lee K, Toutanova K BERT: Pre-training of Deep Bidirectional Transformers for Language Understanding. arXiv.

[40] Zhang Y, Jin R, Zhou ZH (2010). Understanding bag-of-words model: a statistical framework. Int J Mach Learn & Cyber.

[41] Leskovec J, Rajaraman A, Ullman JD (2014). Mining of Massive Datasets, 2nd Edition.

[42] Naseem U, Razzak I, Khan S, Prasad M A Comprehensive Survey on Word Representation Models: From Classical to State-Of-The-Art Word Representation Language Models. arXiv.

[43] Chawla NV, Bowyer KW, Hall LO, Kegelmeyer WP (2002). SMOTE: Synthetic Minority Over-sampling Technique. JAIR.

[44] Jurafsky D, Martin J (2008). Speech and Language Processing, 2nd Edition.

[45] Zhu Y, Kiros R, Zemel R Aligning Books and Movies: Towards Story-like Visual Explanations by Watching Movies and Reading Books. arXiv.

[46] Sun C, Qiu X, Xu Y, Huang X (2019). How to Fine-Tune BERT for Text Classification?. Chinese Computational Linguistics. Vol 11856. Lecture Notes in Computer Science.

[47] (2021). Horizon Scanning Assessment Report? Artificial Intelligence. ICMRA.

[48] (2021). Fostering a European Approach to Artificial Intelligence. European Commission.

