# Supplementary material for: Use of Social Media Data to Diagnose and Monitor Psychotic Disorders: Systematic Review
Source: J Med Internet Res. 2022 Sep 6;24(9):e36986. doi: 10.2196/36986 (PMC9490531; doi:10.2196/36986)
Supplement: Multimedia Appendix 1 [file jmir_v24i9e36986_app1.docx]

Supplementary file 1. Machine learning parameters

Several parameters can be used to evaluate the performance of machine learning algorithms. The result can be represented by a curve of performance characteristics (receiver operating characteristics (ROC) curve). This curve illustrates the relationship between the true positive rate (sensitivity) and the false positive rate (1-specificity). To summarize the overall accuracy of the test, it is possible to calculate the area under the curve (AUC). The range of this value is between 0 and 1. A value of 0.5 indicates an absence of discrimination, a value of 1 indicates perfect precision [23].

The results can also be informed using F1 score, precision and accuracy. This metric calculates the harmonic mean between the precision and recall [24]. Precision is the number of true positives divided by the sum of true positives and false positives where the recall is the number of true positives divided by the sum of true positives and false negatives. Note that the sensitivity and the recall are the exact same metrics. Intuitively the precision gives us information about “how many did we catch” whereas the recall gives us information about “how much did we miss”.

$$precision=\frac{true positives}{true positives+false positives}$$

$$recall=\frac{true positives}{true positives+false negatives}$$

The F1 score is tradeoff between precision and recall given by the formula:

$$F1=2\times\frac{Precision*Recall}{Precision+Recall}$$

As for the AUC, the F1 score is between 0 and 1, 1 indicating a perfect precision and recall.

The main difference between the AUC and the F1 score is that the latter measures precision and recall at any point of the ROC curve separately whereas the former is the area under the curve and thus an average over all possible thresholds. Therefore, one should use the F1 score when the problem has imbalanced classes and avoid the AUC [25].
