# Supplementary material for: Use of Social Media Data to Diagnose and Monitor Psychotic Disorders: Systematic Review
Source: J Med Internet Res. 2022 Sep 6;24(9):e36986. doi: 10.2196/36986 (PMC9490531; doi:10.2196/36986)
Supplement: Multimedia Appendix 2 [file jmir_v24i9e36986_app2.docx]

Supplementary file 2. Search strategy

We based the keywords list on three fields: schizophrenia, AI and social networks. A search strategy was built by using the Booleans operator “AND” and “OR” and applied to titles and abstracts. The keywords and the search strategy were « ((machine learning[Title/Abstract]) OR (deep learning[Title/Abstract]) OR (artificial intelligence[Title/Abstract]) OR (neural network[Title/Abstract])) AND ((schizophrenia[Title/Abstract]) OR (psychosis[Title/Abstract])) AND ((social media[Title/Abstract])OR (facebook[Title/Abstract]) OR (twitter[Title/Abstract]) OR (instagram[Title/Abstract])) » on PubMed and « ((machine Learning) OR (deep learning) OR (artificial intelligence) OR (neural network)) AND (schizophrenia OR psychosis) AND ((social media) OR facebook OR twitter OR instagram) » on EMBASE, PsychINFO and Cochrane in any language, but referenced in the selected databases. On IEE Xplore, we used the keywords “schizophrenia AND machine learning AND social media” applied to title and abstract.
